# Intervening to reduce sedentary behavior among African American elders: the "Stand Up and Move More" intervention

**DOI:** 10.34172/hpp.42548

**Published:** 2024-07-29

**Authors:** Brianna N. Leitzelar, Neda E. Almassi, Susan J. Andreae, Rachelle Winkle-Wagner, Lisa Cadmus-Bertram, Luis Columna, Kevin M. Crombie, Kelli F. Koltyn

**Affiliations:** ^1^Department of Kinesiology, University of Wisconsin-Madison, Madison, WI, 53706, USA; ^2^Social Sciences and Health Policy, Wake Forest University School of Medicine, Winston-Salem, NC 27101, USA; ^3^Department of Educational Leadership and Policy Analysis, University of Wisconsin-Madison, Madison, WI, 53706, USA

**Keywords:** Behavior change techniques, Black or African American, Intervention study, Sedentary behavior

## Abstract

**Background:**

Reducing sedentary behavior is a promising intervention target for improving health for older adults; however, few interventions include African American communities. The purpose of this research was to extend the reach of an effective sedentary behavior intervention to African American elders.

**Methods:**

Two pilot studies assessed the feasibility (retention, adherence, and safety) and acceptability (participant and leader perspectives) of a 4-wk "Stand Up and Move More" (SUMM) intervention. Sedentary behavior (self-reported and monitor-derived), function (short physical performance battery), and quality of life (SF-36) were measured at baseline (wk0), postintervention (wk4), and follow up (wk12; study 1) to examine preliminary effectiveness of the intervention. Participants (N=26) attended SUMM or an attention-matched stress management intervention (study 2). The magnitude of treatment effects were determined using Hedge’s *g* effect size calculations [small (*g*=0.20 to 0.49), moderate (*g*=0.50 to 0.79), large (*g*>0.80)].

**Results:**

Retention and adherence rates ranged from 50%-100% and 80%-100%, respectively. There were no adverse events. Participants expressed high satisfaction, and the leader of the SUMM intervention indicated that the intervention content was beneficial. Hedges’ *g* revealed negligible to small changes in sedentary behavior (*g*<0.50) following SUMM. There were moderate to large improvements in function (*g*=0.51-0.82) and quality of life (*g*=0.54-1.07) from wk0 to wk4 in study 1; and moderate to large improvements in function (*g*=0.51-0.88) from wk0 to wk4 in study 2. There was a moderate improvement in quality of life (SF-36 emotional role limitations *g*=0.54) in the SUMM group only.

**Conclusion:**

Given its feasibility, safety, and acceptability, SUMM may be a promising intervention to improve functioning and well-being among African American elders.

## Introduction

 The proportion of the U.S. population > 65 years is projected to increase from 16% in 2018 to approximately 22% in 2040 and will be the most ethnically and racially diverse older adult population in recorded history.^1^ For instance, the number of older, non-Hispanic White adults is projected to grow by 32% while the number of older African American adults is projected to grow by 88% over the same time period [Note. The term ‘African American’ is being used synonymously with ‘Black’ upon preference from the community partners who were part of this study.] The changing demographic landscape of the U.S. means there is a growing segment of the population who face unique health challenges. Aging is associated with increased risk for early death, multiple chronic diseases (e.g., cardiovascular disease, type 2 diabetes, cancer, etc),^2^ and functional decline,^3-5^ with these health challenges disproportionally affecting African American older adults.^6,7^ Thus, there is a critical need to address health inequities within African American elder communities.

###  Physical function: an urgent health concern among African American elders

 Health inequities span health outcomes, though physical function is particularly important for aging populations. Those who identify as African American face racism and additional systemic barriers at interpersonal, institutional, and structural levels, which negatively impact health and quality of life.^8,9^ African American adults, women in particular, have been shown to age faster^10^ and to have lower life expectancy, fewer years living free of activity limitations, and higher rates of chronic disease^11^ as compared with White adults. Further, African American adults report more functional limitations,^12,13^ are more likely to develop impairments,^14^ and have higher prevalence rates of disability^15,16^ than White adults. Since functional decline is further associated with early mortality and lower quality of life,^17,18^ maintaining or improving physical function is an important intervention outcome.

###  Shifting the focus: sedentary behavior

 Physical activity is an established approach for preserving physical function for older adults,^3^ however, emerging evidence suggests that shifting the focus to reducing sedentary behavior (i.e., prolonged sitting, reclining, and/or lying down) may be a promising alternative strategy since increasing physical activity can be challenging for many older adults.^19^ African American adults report several barriers to adopting a physically active lifestyle such as family demands/lack of time, physical limitations, poor health, fear of falling, and lack of social support, among others.^20-22^ Race-related stressors may also contribute to physical inactivity leading to poor sleep and chronic inflammation, both of which are associated with low activity levels.^8,23^ Furthermore, African American adults > 60 years spend the majority of their day sedentary,^24,25^ which further increases risk for early death,^26^ chronic disease,^27,28^ and functional decline.^29,30^ Shifting the emphasis towards reducing time spent sitting or reclining (i.e., in sedentary behaviors), and replacing it with standing and/or light-intensity physical activities, provides a simple alternative behavior which may have health benefits.

###  Intervening to reduce sedentary behavior: the need for feasibility studies

 More work is needed to understand the feasibility and acceptability of interventions targeting sedentary behavior in African American elder populations. To date, one study demonstrated the feasibility of a 12-week remotely-delivered sedentary behavior intervention (i.e., text messages, newsletters, and telephone coaching) for African American adults; however, no adults > 65 years were included in that study.^31^ Studies from other older adult populations suggest that health coaching (e.g., behavioral feedback, goal setting, self-monitoring)^32-38^ and printed educational materials^39,40^ were feasible and well-liked. Due to the limited amount of research testing sedentary behavior interventions among African American older adults, an important first step is to conduct a feasibility trial in order to inform future, large-scale randomized controlled trials.^31^

###  Study objectives

 The purpose of the current study was twofold: (1) to examine the feasibility and acceptability of an intervention designed to reduce sedentary behavior and (2) to gather preliminary evidence of intervention effectiveness to reduce sedentary behavior and improve physical function and quality of life for African American elders.

## Materials and Methods

 This study was a subset of a larger, community-based randomized controlled trial (Identifier: NCT03412084)) designed to examine the effectiveness and feasibility of translating a sedentary behavior intervention by State Aging Units to older adults residing in four counties in Wisconsin (WI), USA. The present paper describes the research conducted with an African American elder community. Results from the three other counties have been previously published.^41^

**Figure 1:**
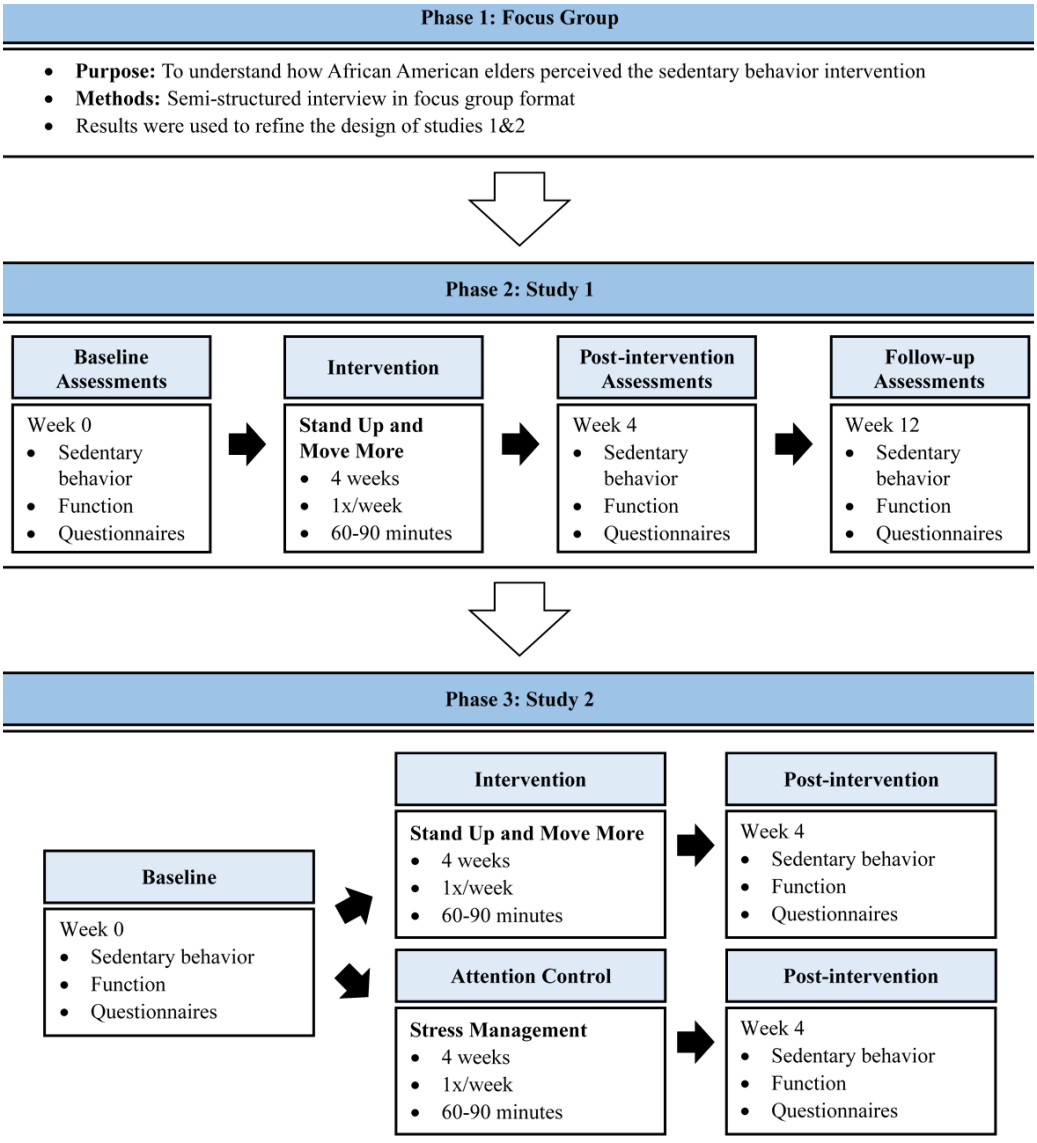
Study Schematic

 The present work included three phases (Figure 1). Phase 1 (“focus group”) was a qualitative study designed to understand how African American elders perceived the sedentary behavior intervention through a semi-structured focus group interview. Phase 2 (“study 1”) was a single-arm pilot study where participants completed assessments at baseline (i.e., week 0), post-intervention (i.e., week 4), and follow-up (i.e., week 12). Participants also provided feedback on the intervention and study assessments. Phase 3 (“study 2”) was a 2-arm randomized controlled trial. Participants were randomly assigned to the sedentary behavior intervention or an attention-matched control group and completed assessments at baseline (i.e., week 0) and post-intervention (i.e., week 4).

###  Community context and partnership

 This research was conducted in collaboration with a local African American community to extend the reach of a novel intervention to reduce sedentary behavior for African American elders. The intervention, “Stand Up and Move More” (SUMM), was originally developed with community-academic partners and pilot tested in one county. Details on the initial community partnership, development, and pilot testing of SUMM can be found elsewhere.^42,43^ For the current study, a community partnership with an African American elder community was established. A trusted member of the African American community (i.e., community leader) was recruited to join the study team, and together, the study team identified a community space which was easily accessible for African American elders for the study. In addition, a health promotion coordinator who identified as African American was hired to recruit participants and to deliver the SUMM intervention. Eligibility criteria for the health promotion coordinator included previous experience delivering health education workshops to older adults from racially and ethnically diverse backgrounds. The health promotion coordinator served as the main point of contact for study participants. She also attended a day-long training session to learn the SUMM curriculum and strategies to facilitate behavior change by empowering older adults to set and achieve their own goals.

###  Focus group

 A focus group session was held at the outset to assess prospective participants’ perceptions of the SUMM workshop and promotional materials in partnership with a survey center on campus with expertise in administering focus group sessions. The community leader recruited 10 African American women between the ages of 55-78 years. During the focus group, a research team member gave a presentation on the content of SUMM and then participants provided feedback regarding their likes, dislikes of SUMM, and made recommendations for enhancing the cultural relevancy of the program and promotional materials (i.e., study flyer). The focus group was audio record, transcribed, and the survey center created a summary report. Results indicated that participants held positive perceptions of SUMM, indicated it would be feasible to attend, and suggested the study flyer should include less text and more pictures of African American elders. It was concluded that no changes were needed to the program content. However, changes were needed to the study flyer (i.e., add representative pictures, edit the text, include community partnerships) to make it more visually appealing. Participant remuneration included a $25 gift card.

###  Study 1

 The initial intent of study 1 was to randomly assign participants to a SUMM workshop group or a wait-list control group. However, there was low acceptability with randomization to the waitlist control group (i.e., participants were wary about waiting 12 weeks to participate in SUMM, resulting in a majority of the waitlist control group attending the introductory session for SUMM instead of their assigned waitlist control session). Upon discussion with the health promotion coordinator, it was decided to combine the two groups and offer the SUMM workshop to everyone in order to build trust with the community. Therefore, study 1 tested the feasibility of the SUMM workshop using a single arm design. Participants completed assessments at baseline (i.e., week 0), post-intervention (i.e., week 4), and follow-up (i.e., week 12) and received a gift card of up to $50 following participation in study 1.

####  Study sample and recruitment of participants

 Sample size was estimated for the larger randomized controlled trial consisting of four counties. A power analysis (G*Power 3.1) was performed to estimate optimal sample size to detect potential differences in monitor-derived sedentary behavior between intervention and wait-list control groups using a repeated measures design with an alpha of 0.05, a power of 0.80, and a moderate effect size based on our pilot study. Results from the power analysis estimated approximately 20 older adults from each county would be needed to participate in this study. The present paper presents findings from only one county (African American elder community).

 The health promotion coordinator recruited participants through word of mouth and by posting flyers in areas accessed by African American elders. Interested individuals contacted the health promotion coordinator who then shared their contact information with the study team on a weekly basis. All interested individuals completed preliminary phone screening with a study team member to determine eligibility. Inclusion criteria included: > 55 years of age, self-identifying as African American, residing in the community where this research was conducted, and living in a home or apartment. Exclusion criteria included: < 6 hours/day of self-reported sedentary behavior (assessed by self-reported sitting time from the day prior to screening), uncontrolled medical conditions (i.e., hypertension, heart disease, actively receiving chemotherapy or palliative care for cancer, stage 4 liver disease, end-stage renal or pulmonary disease), recent hospitalization (i.e., within the past month), severe arthritis or any orthopedic condition that could be made worse by standing up and moving more, the inability to stand up without assistance from anyone else, and the inability to speak/understand English.

####  Intervention description

 The SUMM intervention was delivered by the health promotion coordinator as a small in-person group workshop with sessions held once/week for 4 weeks with a refresher session at 8 weeks. Sessions lasted 1.5-2 hours and were held on the same day and time each week, with the leader providing light refreshments for each session. The SUMM intervention has been previously described.^41,43^ In brief, SUMM was grounded in self-regulation theory^44^ and social cognitive theory^45^ and incorporated several behavior change strategies. Participants learned about sedentary behavior and the benefits of standing up and moving more, engaged in group discussion and brain storming activities, and completed handouts to facilitate personalized goal setting and action planning (i.e., 1-2 strategies to do during the week). Older adults were asked to break up prolonged sitting ( > 1 hour) with short breaks (e.g., get up and move for a couple of minutes multiple times throughout the day) starting with an extra 3-5 times/day from their norm, progressing to 10-12 times/day by the end of the 4-week intervention. Participants used a small click counter to self-monitor the number of times they stood up each day and completed a daily log sheet at home. A refresher session was held at 8 weeks after the initiation of the workshop to encourage maintenance of behavior change (see Crombie et al^41,43^ for further detail). Intervention fidelity was maintained with a “facilitator manual” which included instructions, scripts, and handouts for each session. Moreover, the research team contacted the health promotion coordinator following each session to discuss any deviations from the instruction manual.

####  Procedures

 Prior to initiation of the workshop sessions, participants met with the research team and completed an introductory session, signed an informed consent document, and completed baseline testing which lasted approximately 45 minutes. Baseline testing consisted of a demographic and health history survey, completion of a questionnaire packet, physical function testing, and assessment of sedentary behavior (self-reported and monitor-derived). The same assessments (with the exception of the demographic and health history survey) were repeated immediately following the workshop (i.e., week 4) and at follow up (i.e., week 12).

####  Outcome measures

*Feasibility:* Feasibility was assessed using adherence rates, retention rates, and safety of the SUMM workshop. Adherence rates were calculated using participant attendance at the SUMM sessions (i.e., number of sessions completed). Retention rates were calculated using participant attendance at SUMM assessments (i.e., number of assessment sessions completed). Safety of the program was assessed at the beginning of each workshop session through a “safety questionnaire” which assessed injuries, hospitalizations, and changes in pain.

*Acceptability:* Acceptability of the SUMM workshop was assessed through participant satisfaction. The health promotion coordinator assessed participant satisfaction verbally immediately following the fourth workshop session using the following questions: “*How many thought the workshop was beneficial?*”, “*What did you like about the workshop?*”, “*What did you not like about the workshop?*”, “*What strategies worked the best?”*. She summarized the responses on paper and gave these notes to the research team. Further, to obtain feedback of participants’ satisfaction with the research study methods separate from the SUMM workshop, a member of the research team asked participants the following questions during a one-on-one interview at the week 12 assessments: *“What did you like about the assessments with us?”, “What didn’t you like about the assessments?”, “What do you think we did well?”, “How do you think we can improve?”, *and* “Any more feedback?”*.

*Monitor-derived sedentary behavior: *Sedentary behavior was assessed for 7 consecutive days at each of the assessment sessions using two small activity monitors (i.e., activPAL and ActiGraph). The activPAL (activPAL3^TM^ micro, PAL Technologies Ltd, Glasgow, Scotland) measured horizontal/vertical position of the device (i.e., posture), and was mounted to the midline of the thigh using a hypoallergenic medical dressing. This device measured time spent in sedentary behavior and the number of times sedentary behavior was disrupted by standing up. The ActiGraph (GT3XP-BT, ActiGraph LLC, Fort Walton Beach, FL, USA) recorded the frequency of accelerations during ambulatory activities quantifying physical activity across the entire spectrum (e.g., light intensity, moderate intensity, and vigorous intensity physical activity), and was mounted at the hip using an elastic belt. Participants also kept a paper log of their monitor wear time. Participants had to meet minimum wear time criteria to be considered valid ( > 4 days/week, > 10 hours/day)^46^ and included in analyses. Data were processed using the validated Sojourns Plus Posture method which integrates activPAL and ActiGraph data.^47,48^

*Self-reported sedentary behavior: *The Measurement of Older Adults’ Sedentary Time (MOST) 7-day recall questionnaire in interview format^49^ was used to assess self-reported sedentary behavior. Participants reported the amount of time they spent sitting and participating in 8 different activities (i.e., watching TV, using the computer, reading, socializing, during transport such as driving or while on a bus, while participating in hobbies, while at work or volunteering, and during other activities) each day.

*Function:* Physical function was measured objectively using the Short Physical Performance Battery (SPPB)^50^ which is a brief series of performance tests assessing balance, gait speed, and timed chair stands.

*Questionnaires:* Participants completed a packet of questionnaires at each time point. Health-related quality of life was assessed using the Medical Outcomes Study 36-item Short Form Health Survey (SF-36)^51^ which consisted of 8 subscales: bodily pain, general health, limitations due to physical health, limitations due to emotional health, mental health, physical functioning, social functioning, and vitality. Pain intensity and pain interference were assessed using the Patient-reported Outcomes Measurement Information System (PROMIS) pain intensity short-form and the pain interference-short forms.^52^ The presence of sarcopenia was measured using the Sarcopenia five-item questionnaire (i.e., SARC-F).^53^ Potential psychosocial factors relevant for behavior change were assessed at baseline using the following measures: self-regulation,^54^ self-efficacy,^55^ outcome expectancies,^56^ and habit strength.^57^ However, participants expressed concerns about the high demand of completing questionnaires (i.e., too many of them), thus, only the self-regulation questionnaire was administered during the week 12 assessment. The 12-item self-regulation questionnaire was adapted from the validated Physical Activity Self-regulation Scale^54^to assess self-regulation for increasing the number of stands per day (i.e., standing up and moving more).

####  Statistical analyses

 Feasibility (adherence and retention rates, safety) and acceptability (participant satisfaction) data were summarized using descriptive statistics (i.e., frequencies for categorical variables; means and standard deviations for numerical variables). Due to the preliminary nature of this pilot study and small group format, the magnitude of treatment effects were determined through Hedges’ *g* effect sizes (defined as the mean difference divided by the pooled and weighted standard deviation).^58^ Interpretation of Hedges’ *g* is based on categorizing the magnitude of effect as small (*g* = 0.20 to 0.49), moderate (*g*= 0.50 to 0.79), or large (*g* > 0.80). [Note: analyses of monitor-derived sedentary behavior was restricted to the data obtained from the ActiGraph monitor and processed using the Sojourns method^47^ due to suboptimal wear time of the activPAL monitor and substantial missing data (e.g., only 2 participants had valid data for the week 12 assessment). The Sojourns method utilizes R software for data cleaning and processing (R 3.6.2; https://www.R-project.org/)].

## Study 1 Results

###  Participants

 Ten individuals signed the informed consent document and completed baseline assessments. Eight individuals with a mean age of 69.4 years (standard deviation, SD = 7.8) attended the SUMM workshop. All participants indicated they were female, African American, and not Hispanic or Latino. Further, participants reported an average of 3.3 (SD = 1.4) present health conditions on the demographic and health history questionnaire. The top three reported health conditions were high blood pressure, diabetes, and arthritis. See Table S1 (Supplementary file 1) for the full demographic and health history information for study 1.

###  Feasibility (adherence, retention, and safety)

 Study 1 adherence rate was 80%; eight of those who attended the introductory session completed SUMM (i.e., attended > 3 sessions). The retention rate for completing the assessments at week 4 was 60% (six out of the 10 participants who attended the introductory session completed week 4 assessments), while the retention rate for completing assessments at week 12 was 50% (five participants completed week 12 assessments). There were no study-related adverse events during study 1.

###  Acceptability (participant satisfaction)

 All of the participants (100%) indicated they thought the workshop was beneficial. Participants indicated that they liked SUMM because it was encouraging, there was good conversation, and participants reported liking the group aspect/making new friends. Participants also liked that the workshop included action plans for reaching goals and the presence of snacks at the sessions. Dislikes of the workshop included the types of refreshments provided (e.g., participants wanted coffee in the morning and different snacks), a few participants did not like the time of day the workshop was held (i.e., 9:00 AM), and that the tables and chairs were on wheels. Participants reported the following strategies worked the best to reduce sedentary behavior: dancing, standing while cooking, mindfulness, using stairs with handrails, and standing during TV commercials. Other strategies used included standing up while playing board games, enlisting social support (i.e., from family and friends), setting times on their phone, and putting notes around the house. Participants reported performing the following activities when they stood: dancing, stretching, walking around the house, doing chores, walking more, leaving the house (i.e., for appointments, meetings, socializing), and personal grooming activities.

 Participants also provided feedback on their satisfaction with the research methods separate from the SUMM workshop itself. Some participants felt the research team was supportive and respectful and few changes were needed. Others indicated a lack of rapport with the research team (e.g., the study team was only present to administer assessments which was perceived as exploitative and the researchers were only there to take from the community) and high participant burden (e.g., assessments took too much time and there were too many questionnaires). In addition, participants indicated they did not like wearing the monitors, particularly the activPAL because the hypoallergenic tape used to affix the monitor to the thigh was cumbersome and uncomfortable, resulting in a low rate of wear for the activPAL (25-50%).

###  Baseline sedentary behavior

 Participants spent an average of 9 hours and 50 minutes engaged in sedentary behavior/day (monitor-derived total sedentary time mins/day mean = 590.3, SD = 901.7) and performed an average of 53.7 (SD = 13.8) sit-to-stand transitions/day at baseline. According to the results from the MOST sedentary behavior assessment, participants spent the majority of their sedentary time watching TV (41%), sitting during other activities (i.e., at church, eating meals, relaxing, at appointments) (16%), and sitting while socializing (15%).

###  Magnitude of treatment effects

 Hedge’s *g *effect sizes indicated there were negligible to small changes in sedentary behavior and physical activity (*g* < 0.50) following participation in the SUMM workshop (see Table S2 for a summary of effect size changes for all of the outcome measures). Moderate to large effect size improvements were found for physical function including improvements in balance (*g* = 0.60), chair stands (reduction in time to complete 5 chair stands *g* = -0.82), and the total SPPB score (*g* = 0.54). In addition, moderate to large effect size changes were found for aspects of health-related quality of life (i.e., improvements in social functioning *g* = 1.07 and mental health *g* = 0.64; and fewer limitations due to physical health *g* = 0.94 and emotional health *g* = 0.55) immediately following participation in SUMM. In addition, pain interference decreased moderately (*g* = -0.56) while self-regulation increased to a large degree (*g* = 1.46) following SUMM. At follow-up, there were moderate to large improvements in the monitor-derived number of stands/day (i.e., sit-to-stand transitions, (*g* = 0.93)), light intensity physical activity (*g* = 1.97), and mobility (i.e., gait speed (*g* = -0.75)).

###  Study 2

 Upon completion of the pilot study (study 1), participant feedback on the research study methods was incorporated into the adaptation of the next study, which was a small randomized controlled trial. Adaptations to this study included: (1) replacing the wait-list control group with an attention-matched control group (i.e., stress management workshop) to improve acceptability of randomization^59^; (2) building rapport between participants and the research team beyond administering research assessments (e.g., attending the beginning of the workshop sessions, providing requested refreshments); and (3) reducing participant burden by eliminating several questionnaires and the week 12 assessment. To address feedback regarding the activPAL monitors (i.e., the hypoallergenic dressing was cumbersome and uncomfortable), we offered a fabric thigh sleeve in place of the hypoallergenic dressing to affix the activPAL monitor to the thigh. Further, a second community space was added to increase sample size and reach into the African American community.

 The design of study 2 was a parallel randomized controlled trial where participants completed SUMM or an attention-matched control workshop (i.e., Stress Management) using a 1:1 ratio. Workshop sessions were held once per week for four weeks. Assessments were completed at baseline (i.e., week 0) and post-intervention (i.e., week 4). All participants received up to $50 (i.e., gift card) for participating in study 2.

####  Recruitment of participants and randomization

 A new cohort of individuals was recruited for study 2. As described on page 4, sample size estimates indicated approximately 20 participants were needed to detect potential differences in monitor-derived sedentary behavior between intervention and control groups. The same health promotion coordinator recruited participants for both groups using similar recruitment methods as used in study 1, including word of mouth and posting flyers around the community. Inclusion and exclusion criteria were identical to study 1 and are described previously (page 4). All participants completed a preliminary phone screen conducted by the study team. Eligible participants were allocated to their assigned study group by the same study team member who conducted their phone screen. Participants were allocated to groups based on a random allocation sequence which was generated by an online random sequence generator prior to the initiation of Study 2.

####  Intervention arm

 Participants in the intervention group completed the SUMM workshop (same curriculum as study 1). In brief, participants attended four weekly group sessions lasting 1.5-2 hours held at the same time and on the same day each week. Participants were taught how to appropriately set goals, develop action plans to achieve their goals, self-monitor their behavior, and learned problem solving strategies to overcome barriers to behavior change.

####  Attention-matched control arm

 Participants assigned to the attention-matched control arm completed a stress management workshop called “Taking Care of You: Body, Mind, Spirit,” which was delivered by the same health promotion coordinator who delivered the SUMM workshop.^60^ Participants attended four weekly group sessions lasting 1.5-2 hours held at the same time and on the same day of the week. The stress management workshop was designed to teach participants how stress affects the body; coping techniques; and how to promote positivity, resilience, and balance. Content emphasized mindfulness and used strategies from mindfulness-based stress reduction and positive psychology. It is important to note that there was little mention of physical activity and no mention of sedentary behavior in the curriculum of the stress management workshop. Sessions covered topics such as goal setting, mind-body connection, mindfulness and awareness, mindlessness, dimensions of health and interrelatedness, self-care, positive emotions, identifying priorities, connecting stress and physical health, cognitive restructuring, and increasing resiliency and coping.

####  Procedures

 Prior to initiation of the workshop sessions, participants completed an introductory session, signed an informed consent document, and completed baseline testing. Assessments were completed at baseline and week 4 (i.e., immediately following the workshops, with the refresher session and week 12 follow up assessment eliminated as recommended by the health promotion coordinator and previous participants). The outcome measures used in study 2 and described previously (pages 4-5) included, 1) demographic and health history, 2) feasibility (adherence, retention, and safety), 3) acceptability (participant satisfaction of the SUMM workshop, 4) sedentary behavior (self-reported and monitor-derived), and 5) physical function (i.e., SPPB). Participants also completed a packet of questionnaires similar to study 1 (i.e., health-related quality of life, pain, and sarcopenia), but there were two changes made to the questionnaire packet for study 2. First, with the addition of the stress management workshop, the 10-item Perceived Stress Scale (PSS)^61^ was added to the questionnaire packet. Second, in order to reduce participant burden as requested by previous participants, several questionnaires were removed from the packet including questionnaires assessing self-regulation, self-efficacy, outcome expectancies, and habit strength.

 In addition, perspectives from the health promotion coordinator were added to study 2. The health promotion coordinator shared her perspectives about the acceptability of implementing the SUMM project (studies 1 and 2) during a post-study team meeting. The research team took field notes during this conversation, which served as the data source for this outcome.

####  Statistical Analyses

 Analyses for study 2 were similar to study 1. In brief, feasibility (adherence and retention rates, safety) and acceptability (participant satisfaction, perspectives from the health promotion coordinator) were summarized using descriptive statistics. Hedge’s *g*^58^ was used to characterize the magnitude of treatment effects as small (*g* = 0.20 to 0.49), moderate (*g* = 0.50 to 0.79), or large (*g* = 0.80 and above). [Note. analysis of monitor-derived sedentary behavior was restricted to the data obtained from the ActiGraph monitor and processed using the Sojourns method, which is implemented in R (R 3.6.2; https://www.R-project.org/).^47^ Although we piloted the use of a fabric sleeve as an alternative method to affix the activPAL to the thigh, there were logistical challenges with identifying the correct size and it was deemed impractical to administer the activPAL for the week 4 assessment.]

## Study 2 Results

###  Participants

**Figure 2:**
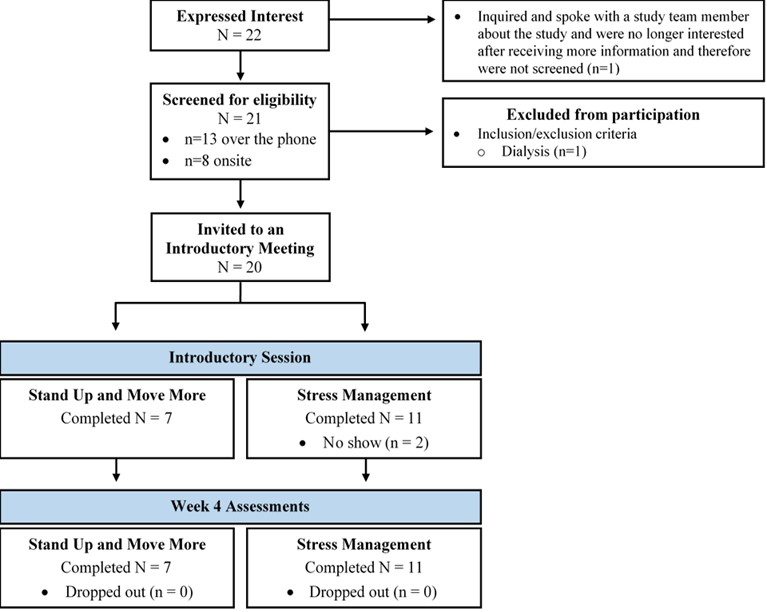
Flow diagram of the progress through study 2

 Eighteen individuals participated in study 2 (Figure 2). Seven individuals completed the SUMM workshop and 11 individuals completed the stress management workshop. The demographic and health information of these participants are summarized in Table S1.

###  Feasibility (adherence, retention, and safety)

 All (n = 7) participants assigned to the SUMM group in study 2 completed the SUMM workshop (SUMM adherence rate = 100%). Adherence data for the stress management group is unavailable because attendance was only collected for 2 out of the 4 stress management workshop sessions. The study 2 retention rate was 100% as all eighteen participants completed the week 4 assessments. There were no study-related adverse events during study 2.

###  Acceptability (participant satisfaction, perspectives from the health promotion coordinator)

 The majority of participants in both groups reported their workshops to be beneficial. Specifically for the SUMM workshop, participants indicated they liked the increased awareness of moving more and the social aspect (i.e., good conversations) of the workshop. Participants did not report any dislikes of the SUMM workshop. The strategy which worked the best for reducing sedentary time was to stand up during TV commercials. Other strategies that worked included: setting a timer to stand up and standing while talking on the phone. When they stood up, participants reported standing to get a drink of water, go to another room, perform a household chore, and/or walk around the house.

 The health promotion coordinator indicated that the SUMM workshop was acceptable to implement. Specifically, she indicated that the community-academic space was a good location for the workshop and described she felt the presence of the research team at the beginning of each workshop session was helpful in building trust. The health promotion coordinator indicated she believed the SUMM workshop to be beneficial for the participants, and thought the workshop, increased participants’ awareness of their sedentary time, felt there were good conversations, and stated the group aspect was an important part of the SUMM workshop. She also indicated that study 2 went better because there was no refresher session nor week 12 assessments.

###  Baseline sedentary behavior

 At baseline, participants across groups spent an average of 11 hours and 35 minutes/day sedentary (monitor-derived total sedentary time mins/day mean = 711.6, SD = 153.6) and performed an average of 41.0 (SD = 15.3) sit-to-stand transitions/day. According to results from the self-report sedentary behavior assessment, the largest proportion of their sedentary time was spent watching TV (32%). Participants also spent 19% of their sedentary time socializing and 16% doing other activities such as eating meals, going to church, and going to appointments.

###  Magnitude of treatment effects

**Figure 3:**
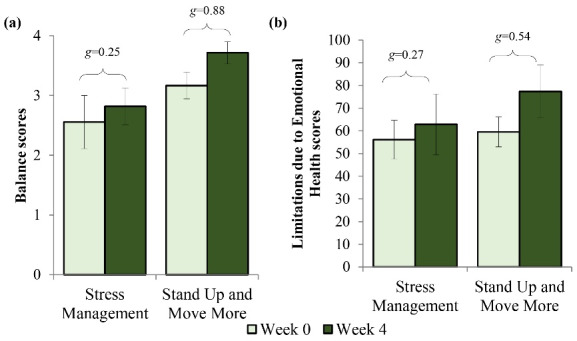
Means, standard errors, and Hedges’ g effect sizes for outcomes that exhibited moderate to large improvements following the SUMM workshop during study 2. (**a**) There was a small improvement in SPPB balance scores following stress management and large improvement following SUMM. (**b**) There was a small improvement in SF-36 limitations to emotional health following stress management and a moderate improvement following SUMM. Higher scores indicate fewer limitations

 There were negligible to small effect size changes in monitor-derived sedentary behavior following participation in both workshops (*g* < 0.50). Other outcome measures that exhibited small effect size changes following both workshops are summarized in Supplementary Table S3. For moderate to large effect size changes, there was a large improvement in the SPPB balance score (*g* = 0.88) for the SUMM group compared to a small change in the stress management group (*g* = 0.25) (see Figure 3a). Also, there was a moderate improvement (i.e., increased score) in SF-36 limitations due to emotional health (*g* = 0.54) for the SUMM group in comparison to a small change (*g* = 0.27) in the stress management group (see Figure 3b).

## Discussion

 There is a strong need for culturally sensitive evidence-based health workshops for African American communities.^62^ The SUMM workshop contributes to filling this need by focusing on a simple health behavior (i.e., standing more) which may have health benefits. To the authors’ knowledge, SUMM is the first group-based intervention to target reductions in prolonged bouts of sitting ( > 60 minutes) among African American older adults ( > 55 years) using self-regulation and social-cognitive theories. Participants incorporated simple strategies to stand up and move more such as standing up during TV commercials, while playing board games, putting reminders on their phone or around the house, and enlisting social support. Two studies were conducted to examine the feasibility, acceptability, and to gather preliminary evidence of intervention effectiveness of the SUMM workshop for African American elders.

 The results from the two studies presented in this article demonstrated SUMM was feasible and acceptable to implement within a community of African American elders. There were no study-related adverse events nor increases in pain during study 1 nor study 2. Adherence rates were excellent (study 1 adherence rate = 80%; study 2 adherence rate = 100%); and it is important to note that taxi rides were provided for participants, which may have contributed to the high adherence rates. The health promotion coordinator indicated SUMM was beneficial for participants and did not think major changes were needed to the content of SUMM. Participants expressed high satisfaction with the SUMM workshop. Content of the workshop included group discussions, setting incremental goals, making specific plans to achieve goals (i.e., action planning), monitoring sedentary behavior, and problem-solving activities. Previous research also indicates the acceptability of such strategies to reduce sedentary behavior in older adult populations.^31-40^

 Results from the magnitude of treatment effects indicate the potential benefits of participating in the SUMM workshop for African American elders. There were moderate to large improvements in physical function (i.e., study 1: improvements in balance, rising from a chair, the total SPPB score; and study 2: improvements in balance) following participation in SUMM. In addition, there were moderate to large improvements in aspects of health-related quality of life (study 1: improvements in mental health, social functioning, and fewer limitations due to physical and emotional health; and study 2: fewer limitations due to emotional health). These results align with the other study in this area^31^ which found moderate improvements in health-related quality of life following a 12-week sedentary behavior intervention in a sample of African American adults with MS who were < 55 years old. Results from the current study extend these findings and suggest that improvements in health-related quality of life can occur in as little as 4 weeks.

## Considerations for future research

 The importance of developing community partnerships and building trust was clear throughout the SUMM project. There is a long history of medical violence enacted by White individuals on African American bodies, a legacy that still persists today.^6,8,9^ It is suggested that researchers consider this sociopolitical context and build trust with communities. Trust building is critical for community-based research regardless of one’s racial identity; however, may be particularly salient as predominantly White researchers working with an African American community.^62,63^ Trust-building strategies used during the SUMM project included conducting a focus group to understand community perceptions of SUMM, incorporating participant feedback, and attending the beginning of workshop sessions to build rapport with participants. A community feedback dinner was also held following study 2 to celebrate completion of the workshops. Participants received their individual sedentary behavior results and the research team gave an overview of the overall results from the SUMM project. During the evening, several participants indicated they appreciated that there was more than one study conducted in their community and that it was apparent that the health promotion coordinator was a respected member of the study team.

 Key components of community-based research also include aligning research with community interests and centering perspectives of the community throughout the research process.^62^ In the current studies, the participants were asked to provide feedback on both the SUMM workshop and research study methods. Participant feedback was discussed with the health promotion coordinator, and together, we decided how to best align the studies with community perspectives. For example, there was low acceptability of the waitlist control group so the waitlist control was adjusted to an attention-matched control group to enhance acceptability of randomization for study 2.^59^ The topic of the workshop was chosen based on the health promotion coordinator suggestion that a stress management workshop would align with the interests of the African American elder community. Other adaptations for study 2 included providing requested refreshments and reducing participant burden when methodologically possible (see page 6). To reduce participant burden, there was less paperwork (i.e., fewer questionnaires to complete) and the refresher session and follow-up assessments were eliminated in study 2. Further, participants in study 1 expressed their dissatisfaction with wearing the activPAL monitor, resulting in low wear time and missing data. Thus, an alternative strategy (i.e., fabric sleeve) was used in study 2 to affix the activPAL monitor to the thigh, but this method also proved to be unsatisfactory. Challenges with activPAL wear are not unique to African American elders,^64,65^ and future research is needed to develop more practical and acceptable methods to ensure activPAL wear.

## Strengths and limitations

 Strengths of this study included working with an intended audience who are typically underrepresented in health research, the development of community partnerships, incorporation of participant feedback, and inclusion of objective measurements of behavior and physical function. The information gained during this study provide essential insight for the planning and implementation of a future, large-scale randomized trial. Some limitations should also be noted. Male African American elders were underrepresented in this work, thus, it is unclear whether results generalize to male African American elders. In addition, participant feedback of the SUMM workshop was collected by the health promotion coordinator and participants may not have been comfortable sharing negative feedback with the individual leading their workshop. Further, due to the suboptimal wear time of the activPAL monitor, the sedentary behavior data were assessed and processed using only the ActiGraph monitor and Sojourns method. There is some research suggesting that the Sojourn method using ActiGraph data is a reasonable method for estimating sedentary behavior; however, due to the hip-worn placement, it is less capable of differentiating between sedentary activity (i.e., sitting) and light-intensity activities (i.e., standing, moving) than the Sojourn Plus Posture method which incorporates both activPAL and ActiGraph data.^47,48^ Lastly, seven individuals who participated in study 2 were not able to be randomized due to unforeseen circumstances (e.g., participants attended the introductory session for the stress management workshop without the prior knowledge of the study team and could not be randomized), thus true randomization was not achieved for study 2.

## Conclusion

 This study identifies the potential benefit of the SUMM workshop for African American elders. The SUMM workshop was feasible, safe, and acceptable for participants. The health promotion coordinator found the workshop to be feasible and acceptable to implement. Further, there were consistent moderate to large improvements in physical function and health-related quality of life following participation in the SUMM workshop. Further research with larger sample sizes is required to replicate these results. In conclusion, the results from these studies suggest that the SUMM workshop is safe and beneficial and could serve as an important intervention to improve the functioning and well-being of older adults who identify as Black or African American.

## Author Note

 Two authors moved institutions during the writing of this manuscript. Neda E. Almassi is currently affiliated with the Department of Neurology at the University of Pennsylvania. Kevin M. Crombie is currently affiliated with the Department of Kinesiology at The University of Alabama.

## Acknowledgements

 The authors would like to thank all the participants for their time and interest in this study; Fabu Carter for her partnership, advice, and facilitation of the focus group; Pam Bracey for her insight, work with recruitment, and facilitating the workshops; Kelly Elver from the UW Survey Center who developed the focus group interview guide and facilitated the focus group; the UW South Madison Partnership Office for providing the main location for the study; Jill Renken for leading the training session on delivering the “Stand Up and Move More” curriculum; and our lab assistants (Allison Steube, Katie Knott, and Samantha Dawes) for assisting with data entry.

## Competing Interests

 None.

## Ethical Approval

 Human subjects approval was obtained from the University of Wisconsin-Madison Education and Social/Behavioral Sciences Institutional Review Board (IRB; protocol number 2013-1155). All participants provided written informed consent to participate. The protocol for the Stand Up and Move More parent study was pre-registered on ClinicalTrials.gov (Identifier: NCT03412084).

## Supplementary Files


Supplementary file 1 contains Tables S1-S3.
