# Supplementary material for: Intervening to reduce sedentary behavior among African American elders: the "Stand Up and Move More" intervention
Source: Health Promot Perspect. 2024 Jul 29;14(2):148–60. doi: 10.34172/hpp.42548 (PMC11403339; doi:10.34172/hpp.42548)
Supplement: Supplementary file 1 — contains Tables S1-S3. [file hpp-14-148-s001.pdf]

Leitzelar et al, **Health Promotion Perspectives,** 2024, 14(2), 148-S1
doi: 10.34172/hpp.42548
https://hpp.tbzmed.ac.ir

Supplementary file 1

Table S1. Demographic and health history information – Study 1 and Study 2

**Variable**	**Study 1** n=8	**Study 2** SUMM n=7	Stress Management n=11
**Age** (years)	71.7 ± 8.4	63.6 ± 8.8	75.3 ± 5.9
**BMI** (kg/m^2)	28.9 ± 9.7	30.8 ± 9.9	26.1 ± 6.8
**Health Rating** (range 1 to 5)	2.7 ± 1.0	3.3 ± 0.5	3.0 ± 0.5
**Sex** (% female)	100%	86%	82%
**Race** (% of sample)			
African American	100%	100%	82%
Multiracial	0%	0%	18%
**Ethnicity** (% of sample)			
Not Hispanic or Latino	100%	71%	27%
Did not answer	0%	29%	73%
**Educational Attainment** (% of sample)			
High School	38%	29%	27%
College	50%	57%	46%
Graduate	0%	14%	18%
Missing	12%	0%	9%
**Marital Status** (% of sample)			
Divorced	13%	29%	0%
Married	0%	43%	18%
Single	63%	29%	36%
Widowed	25%	0%	46%
Missing	13%	0%	0%
**Present Health History** (% yes)			
Arthritis	75%	100%	82%
Cancer	13%	0%	9%

Cardiovascular disease	13%	0%	18%
Chest discomfort	13%	14%	18%
Diabetes	88%	43%	36%
Dizzy spells	0%	14%	27%
Falls	0%	43%	27%
Heart palpitations	0%	0%	9%
High Blood Pressure	63%	86%	55%
Hip or knee replacement	13%	0%	9%
Orthopedic Problems	13%	57%	18%
Stroke	13%	0%	0%
Other [a]	0%	0%	18%
**Current Smoker** (% yes)	13%	0%	0%
**Current Caffeine Use** (% yes)	38%	57%	64%
**Current Alcohol Use** (% yes)	13%	0%	0%

Note. BMI=Body Mass Index
[a]other present health conditions included asthma, chronic kidney disease stage 3, and the presence of a heart defibrillator

Table S2
Means, standard deviations, and effect sizes for all effectiveness outcomes following the SUMM workshop– study 1

Outcomes	Week 0		Week 4		Week 12		Week 0 to Week 4	Week 0 to Week 12
	n	M ± SD	n	M ± SD	n	M ± SD	Hedges' *g*	Hedges' *g*
Self-reported Total Sedentary Time (mins/day)	6	539.1 ± 183.7	6	473.3 ± 218.2	5	469.3 ± 137.5	-0.33	-0.42
Sit-to-Stand Transitions	5	53.7 ± 13.8	5	50.2 ± 8.1	2	66.0 ± 10.4	-0.31	0.93
Monitor-derived Total Sedentary Time [illegible]	5	590.3 ± 90.7	5	582.7 ± 101.2	2	711.8 ± 76.3	-0.08	1.38
Light Intensity Physical Activity (mins/day)	5	989.0 ± 12.3	5	94.1 ± 18.8	2	121.3 ± 5.8	-0.30	1.97
MVPA (mins/day)	5	53.2 ± 66.7	5	43.3 ± 39.6	2	35.4 ± 2.0	-0.18	-0.30
Total SPPB Score	6	7.0 ± 2.8	6	8.5 ± 2.8	4	8.0 ± 0.8	0.54	0.45
SPPB Balance Score	6	2.8 ± 1.5	6	3.5 ± 0.6	4	3.3 ± 1.0	0.60	0.32
SPPB Gait Speed (m/s)	6	1.5 ± 0.4	6	1.4 ± 0.4	4	1.2 ± 0.2	-0.16	-0.75
SPPB Timed Chair Stand Test (s)	4	16.0 ± 2.8	5	14.0 ± 2.0	3	16.2 ± 5.7	-0.82	0.06

Physical Function	4	61.3 ± 30.9	5	65.0 ± 24.7	5	43.0 ± 24.7	0.14	-0.66
Limitations due to Physical Health	6	55.2 ± 16.0	6	71.9 ± 19.3	5	57.5 ± 22.3	0.94	0.12
Limitations due to Emotional Health	5	63.3 ± 28.0	6	77.8 ± 25.1	5	45.0 ± 22.5	0.55	-0.72
Vitality	6	56.3 ± 19.4	6	63.5 ± 20.3	5	61.3 ± 13.6	0.37	0.29
Mental Health	6	58.3 ± 24.6	6	72.5 ± 19.7	5	67.0 ± 25.2	0.64	0.35
Social Functioning	6	60.4 ± 34.8	6	89.6 ± 16.6	5	62.5 ± 34.2	1.07	0.06
Bodily Pain	6	69.6 ± 24.2	6	79.3 ± 22.4	5	63.0 ± 35.8	0.42	-0.22
General Health	6	69.2 ± 10.8	6	72.8 ± 10.2	5	75.6 ± 12.8	0.35	0.55
Sarcopenia	5	3.2 ± 3.6	6	2.0 ± 2.7	5	2.8 ± 2.3	-0.39	-0.13
Current Pain Intensity	6	2.0 ± 1.1	6	1.7 ± 0.8	5	2.0 ± 1.0	-0.35	0.00
Pain Interference	5	10.0 ± 4.1	6	7.5 ± 4.8	4	10.8 ± 5.1	-0.56	0.16
Self-Regulation	4	30.8 ± 12.8	4	46.2 ± 8.5	5	42.6 ± 8.7	1.46	1.11

Note. Self-reported total sedentary time was determined using the sedentary behavior interview. Sit-to-stand transitions, monitor-derived total sedentary time, light intensity physical activity, and MVPA were derived from ActiGraph accelerometer. Balance, gait speed, and timed chair stands were measured using the Short Physical Performance Battery. Physical function, limitations due to physical health, limitations due to emotional health, vitality, mental health, social functioning, bodily pain, and general health scores were obtained from the SF-36. Higher ratings are indicative of higher health-related quality of life. Sarcopenia scores were obtained from the SARC-F. Current pain intensity and pain interference were obtained from the PROMIS pain intensity and interferences forms, respectively. Self-regulation was obtained from a 12-item physical activity self-regulation questionnaire adapted for sedentary behavior. An increase in self-regulation score indicates increased use of self-regulatory strategies to stand up and move more. m/s=meters per second; mins= minutes; MVPA=moderate-to-vigorous physical activity; s=seconds; SPPB=short physical performance battery

Table S3

Means, standard deviations, and effect sizes for all effectiveness outcomes following the Stand Up and Move More and stress management workshops – Study 2

	Week 0		Week 4		
	n	M ± SD	n	M ± SD	Hedges' g
Self-reported Total Sedentary Time (mins/day)					
Stand Up and Move More	6	480.5 ± 161.9	7	415.8 ± 130.8	-0.44
Stress Management	8	598.8 ± 315.5	8	626.4 ± 78.7	0.12
Sit-to-Stand Transitions					
Stand Up and Move More	5	48.6 ± 12.8	5	48.3 ± 7.7	-0.03
Stress Management	8	36.3 ± 15.4	10	36.3 ± 11.8	0.00

Monitor-derived Total Sedentary Time (mins/day)					
Stand Up and Move More	5	648.1 ± 128.1	5	668.1 ± 108.1	0.17
Stress Management	8	751.2 ± 162.5	10	701.12 ± 111.4	-0.37
Light Intensity Physical Activity (mins/day)					
Stand Up and Move More	5	94.9 ± 51.7	5	84.6 ± 25.5	-0.25
Stress Management	8	71.1 ± 46.3	10	84.1 ± 57.6	0.25
MVPA (mins/day)					
Stand Up and Move More	5	24.2 ± 15.6	5	24.2 ± 10.3	0.00
Stress Management	8	9.9 ± 5.5	10	12.6 ± 7.5	0.40
Total SPPB Score					
Stand Up and Move More	6	7.5 ± 3.1	7	7.6 ± 1.9	0.03
Stress Management	9	6.8 ± 3.0	11	6.8 ± 2.2	0.02
SPPB Balance Score					
Stand Up and Move More	6	3.2 ± 0.8	7	3.7 ± 0.5	0.88
Stress Management	9	2.6 ± 1.3	11	2.8 ± 0.8	0.25
SPPB Gait Speed (m/s)					
Stand Up and Move More	6	1.8 ± 1.3	7	1.5 ± 0.6	-0.34
Stress Management	9	1.5 ± 0.3	11	1.5 ± 0.4	0.12
SPPB Timed Chair Stand Test (s)					
Stand Up and Move More	4	14.5 ± 4.1	4	16.1 ± 4.9	0.35
Stress Management	6	21.7 ± 18.0	7	15.5 ± 3.1	-0.51
Physical Function					
Stand Up and Move More	7	42.9 ± 30.9	7	53.6 ± 38.9	0.30
Stress Management	10	48.0 ± 22.1	11	43.6 ± 17.6	-0.22
Limitations due to Physical Health					
Stand Up and Move More	7	56.3 ± 35.2	7	64.3 ± 30.1	0.25
Stress Management	10	46.9 ± 22.9	11	54.6 ± 16.6	0.39
Limitations due to Emotional Health					
Stand Up and Move More	7	59.5 ± 35.5	7	77.4 ± 30.7	0.54
Stress Management	11	56.1 ± 28.4	11	62.9 ± 21.9	0.27
Vitality					
Stand Up and Move More	7	62.5 ± 21.7	7	64.3 ± 16.4	0.09
Stress Management	11	54.6 ± 17.0	11	59.7 ± 17.3	0.30
Mental Health					
Stand Up and Move More	7	74.3 ± 20.1	7	79.3 ± 14.3	0.29
Stress Management	9	72.2 ± 19.4	11	74.6 ± 17.7	0.13
Social Functioning					

Stand Up and Move More	7	58.9 ± 32.0	7	71.4 ± 18.7	0.48
Stress Management	11	72.7 ± 22.9	11	65.9 ± 27.4	-0.27
Bodily Pain					
Stand Up and Move More	7	56.6 ± 23.6	7	46.8 ± 23.1	-0.42
Stress Management	11	52.9 ± 25.7	11	47.8 ± 20.2	-0.22
General Health					
Stand Up and Move More	7	67.1 ± 13.9	7	65.7 ± 17.1	-0.09
Stress Management	9	61.6 ± 20.5	11	60.6 ± 18.8	-0.05
Sarcopenia					
Stand Up and Move More	7	2.6 ± 2.0	7	2.4 ± 2.1	-0.07
Stress Management	10	3.3 ± 2.4	11	4.2 ± 2.8	0.34
Current Pain Intensity					
Stand Up and Move More	7	2.3 ± 1.0	7	2.1 ± 0.9	-0.15
Stress Management	10	1.9 ± 1.0	11	2.2 ± 1.0	0.29
Pain Interference					
Stand Up and Move More	7	10.6 ± 4.0	7	10.4 ± 5.7	-0.03
Stress Management	9	10.9 ± 4.7	11	10.3 ± 4.2	-0.14
Perceived Stress					
Stand Up and Move More	7	15.0 ± 4.3	7	15.4 ± 3.0	0.12
Stress Management	10	17.3 ± 8.0	11	14.3 ± 5.2	-0.45

Note. Self-reported total sedentary time was determined using the sedentary behavior interview. Sit-to-stand transitions, monitor-derived total sedentary time, light intensity physical activity, and MVPA were derived from ActiGraph accelerometer. Balance, gait speed, and timed chair stands were measured using the Short Physical Performance Battery. Physical function, limitations due to physical health, limitations due to emotional health, vitality, mental health, social functioning, bodily pain, and general health scores were obtained from the SF-36. Higher ratings are indicative of higher health-related quality of life. Sarcopenia scores were obtained from the SARC-F. Current pain intensity and pain interference were obtained from the PROMIS pain intensity and interferences forms, respectively. Perceived stress was obtained from the Perceived Stress Scale.
m/s=meters per second; MVPA=moderate-to-vigorous physical activity; s=seconds; SPPB=Short Physical Performance Battery
